# Supplementary figures and images for: Diverse task-driven modeling of macaque V4 reveals functional specialization towards semantic tasks
Source: PLoS Comput Biol. 2024 May 23;20(5):e1012056. doi: 10.1371/journal.pcbi.1012056 (PMC11115319; doi:10.1371/journal.pcbi.1012056)

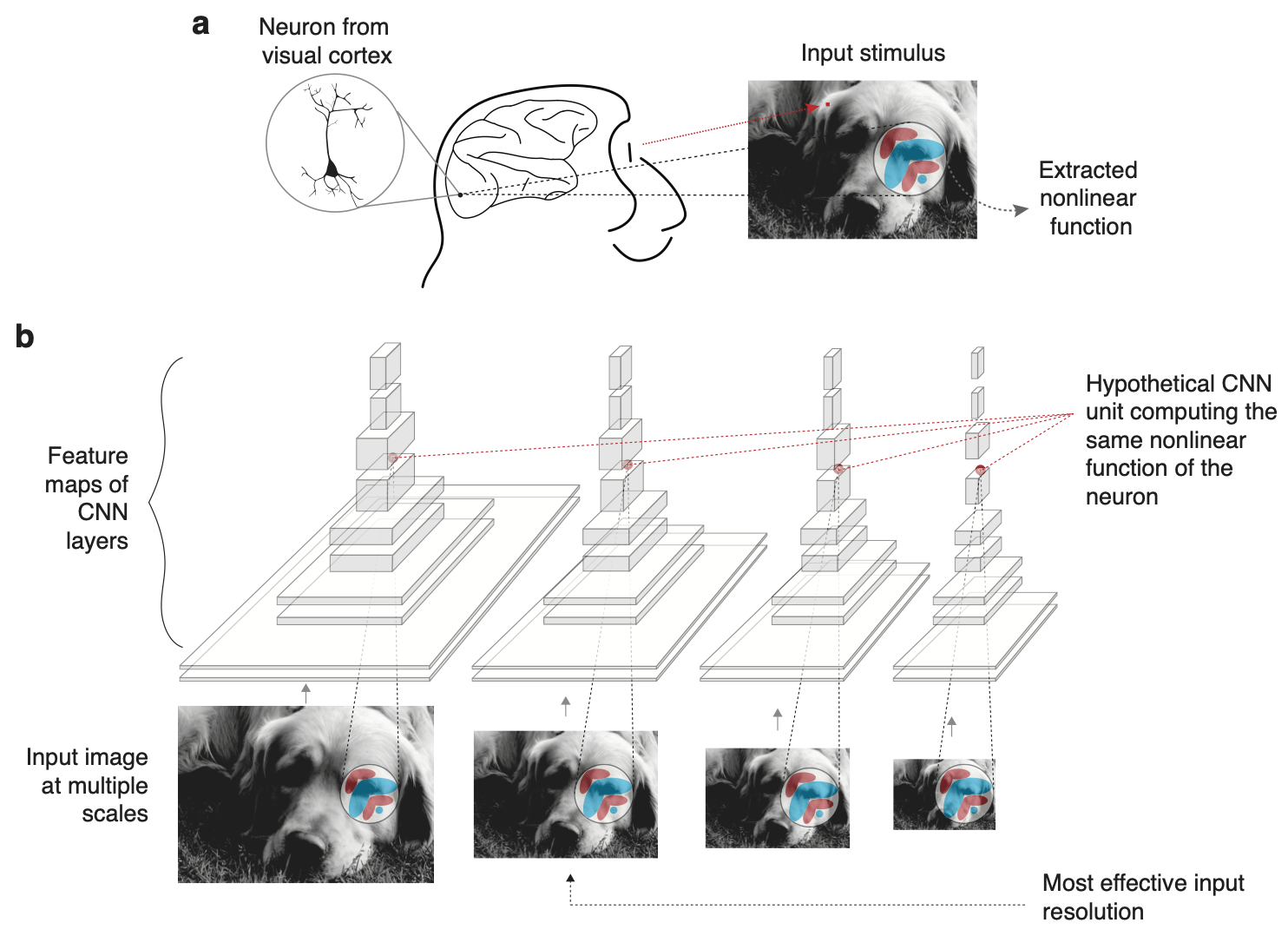

Supplement: S1 Fig — a, A single excitatory neuron from visual cortex, recorded from a head-anchored monkey sitting at a certain distance from a screen and fixating on a spot; extracts a nonlinear function of the input stimulus with a specific receptive field coverage. b, A pretrained deep convolutional neural network (CNN) extracts several nonlinear feature maps at each of its intermediate layers. A single output unit of a feature map computes a nonlinear function on its analytical receptive field with a fixed size in pixels. Even if the real neuron’s nonlinear function was exactly matched to that of a CNN unit, we would have troubles finding it if we were to forward the input image at the wrong input resolution (in terms of pixels per visual angle). It is oftentimes difficult to predict a priori the optimal resolution at which a certain layer extracts the right nonlinearities that best match our responses, especially when the receptive field sizes of neurons are difficult to estimate for higher visual areas, and when recording beyond the foveal region of the visual field. We thus treated the input resolution as a hyperparameter that we cross-validate on the validation set. This facilitates removing the confound between the degree of nonlinearity and receptive field growth when trying to establish hierarchical correspondence between CNN layers and the biological visual system. (TIFF) [file pcbi.1012056.s001.tiff]

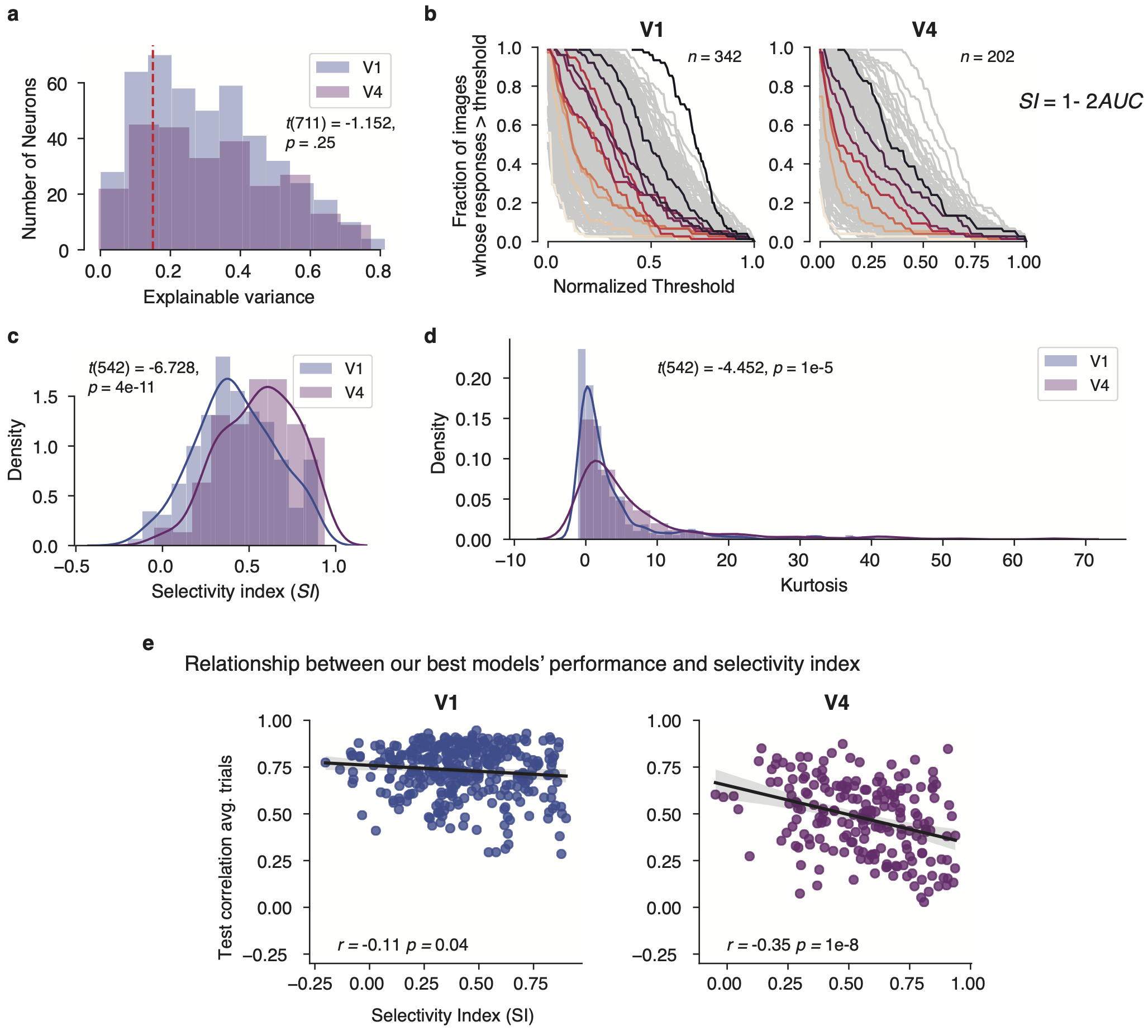

Supplement: S2 Fig — a, Explainable variance (Eq 3) distribution of V1 (n = 458) and V4 (n = 255) neurons. Red line shows the threshold (0.15) we chose to filter unreliable neurons from our test evaluations. b, Curves of the fraction of images evoking responses larger than a threshold vs. threshold value (normalized). We selected 100 evenly spaced thresholds between minimum and maximum value of the responses. Results for 342 neurons in V1 (left) and 202 neurons in V4 (right) show each neuron’s curve (gray). A small sample of curves were colored for clarity. We then computed for each neuron the selectivity index (SI) [24] as 1 − 2AUC where the AUC is the area under the curve. c, Density distribution of selectivity indices in V1 and V4. Two-sided t-test shows that means are different between areas. d, Density distribution of kurtosis statistic computed for each neuron over the test images in V1 and V4. Two-sided t-test shows that means are different between areas, highlighting increase sparsity in V4. e, We evaluated how well SI correlates with the predictive performance of our best model on each area (using features of Robust Resnet50) and found that selectivity index only weakly explains V1 and V4 test performance. (TIFF) [file pcbi.1012056.s002.tiff]

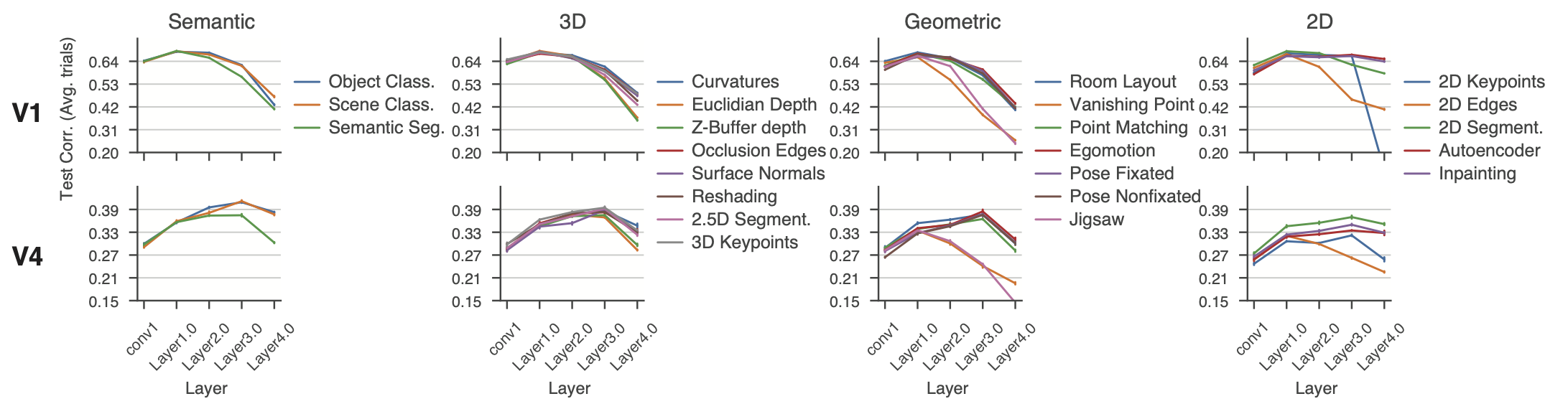

Supplement: S3 Fig — The task-model labels are shared between V1 and V4, and placed to the right of each column. Each line represents the average performance over seeds of the mean performance over neurons of the best task-model configuration in the validation set. That means that these lines represent the test set performance after pooling over input scales, and hyper-parameters (i.e. regularization penalty). Bars represent 95% confidence intervals of 1 s.e. of the mean for five seeds. We measured performance as the average test score over single units (nV1 = 458, nV4 = 255) calculated as the correlation between model predictions and mean responses over repetitions. (TIFF) [file pcbi.1012056.s003.tiff]

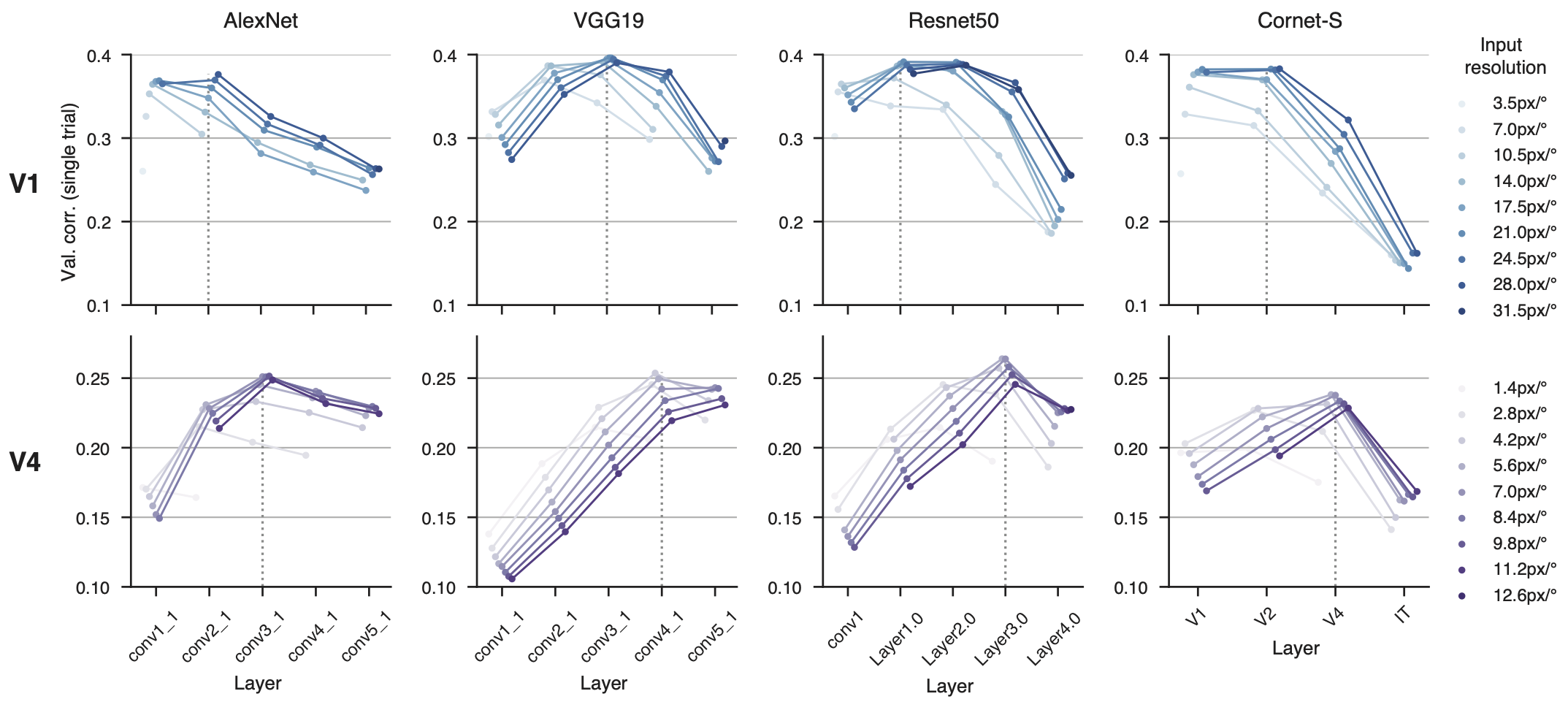

Supplement: S4 Fig — We considered four popular CNNs with different architectures, pretrained on ImageNet (columns; from left to right: AlexNet [29], VGG-19 [30], Resnet50 [20], Cornet-S [31]). For each network, we built neural predictive models that use features from multiple layers (x axis) that span the depth of the network. Each dot in the plot is the average over seeds of the best model configuration pooled over regularization parameters (see Methods). Assigning a layer to a brain area depends on the input scale—the peak of the curves shifts across input resolutions. Moreover, optimizing the layer using the wrong input resolution may lead to sub-optimal performance (S1 Fig). We found that all of these models reveal a hierarchical ordering of nonlinear computations in the two areas, even when we account for input scale—V1 is predicted always by an earlier layer than V4 (dotted vertical lines represent the most predictive layer over scales). (TIFF) [file pcbi.1012056.s004.tiff]

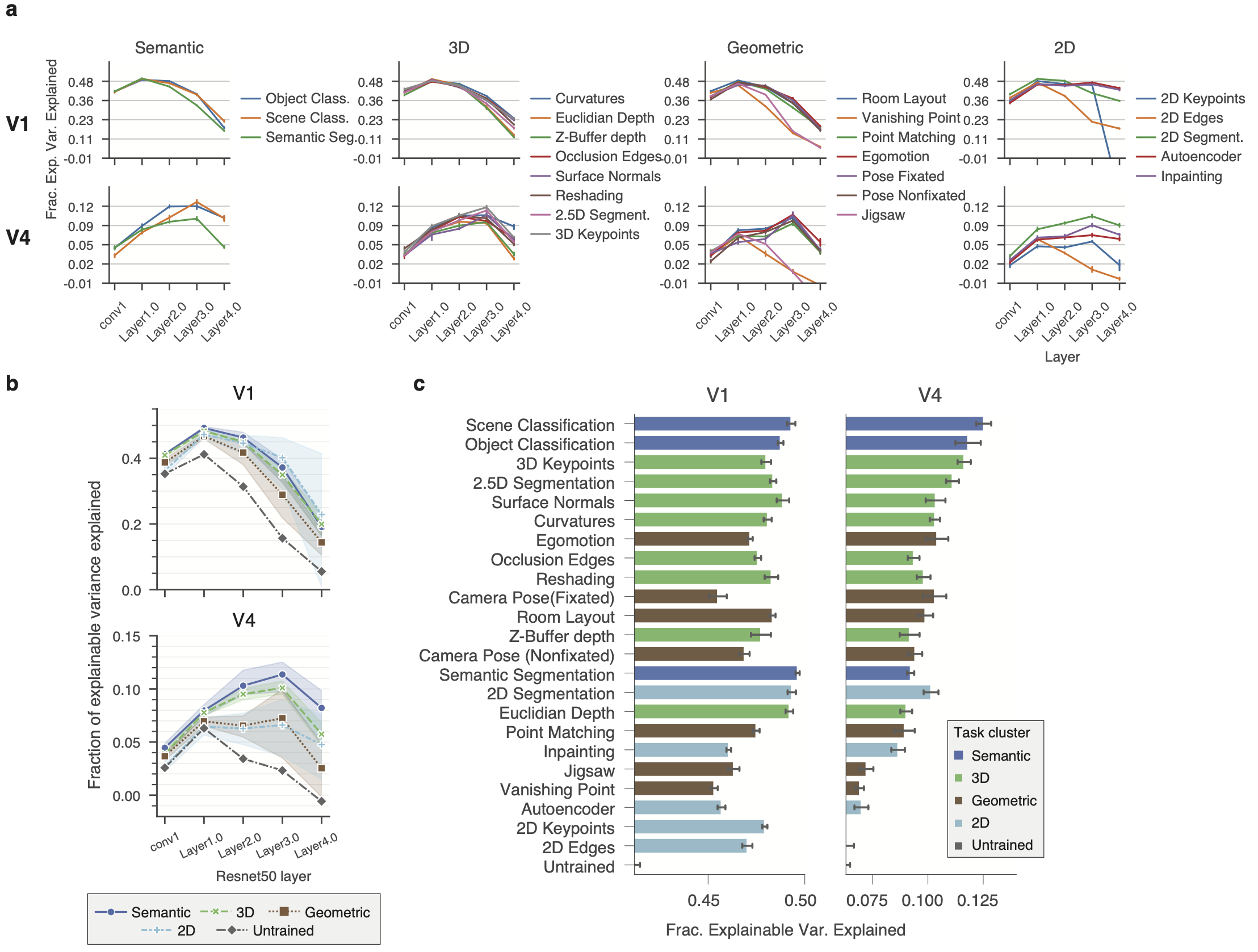

Supplement: S5 Fig — a, Individual task-model performances on V1 (upper row) and V4 (bottom row) as a function of network layer organized in columns by the task-clusters [15]. Equivalent to S3 Fig but performance is measured in terms of FEVE. b, Comparison of diverse task-driven models on V1 and V4 measured in FEVE (Fig 2A and 2B). c, Tasks performances in terms of FEVE after optimizing over layers and hyperparameters on the validation set ordered as in Fig 2C. (TIFF) [file pcbi.1012056.s005.tiff]

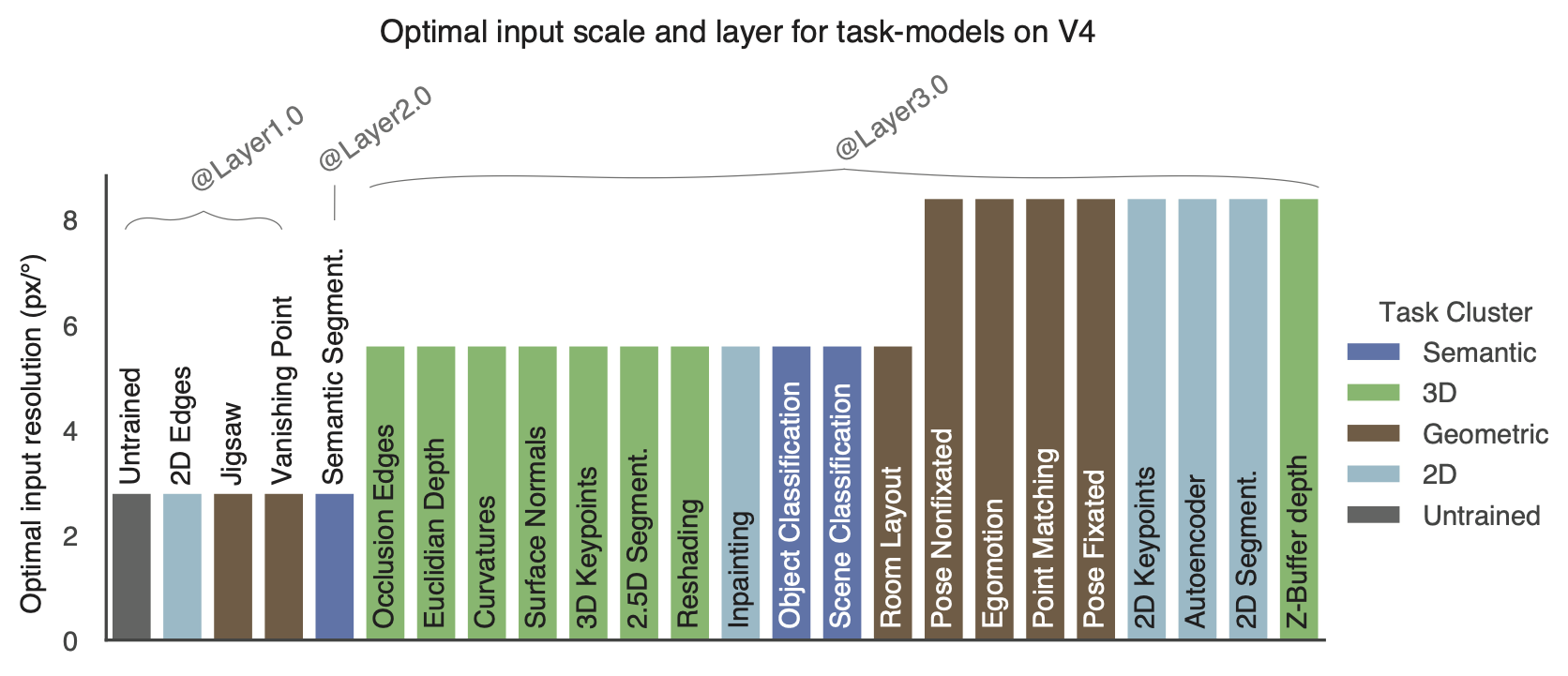

Supplement: S6 Fig — In contrast to area V1 where the optimal layer and scale was shared among all task-models (Layer1.0 and 21px/°), there was variability of the optimal layer in the V4 task-models. In some models, including the untrained network, Layer1.0 with a low input resolution was optimal. The top performing models, including the two semantic classification, and most of 3D tasks (Fig 2C) chose an intermediate resolution (∼ 5.6px/°) at Layer3.0. Interestingly, most geometric and 2D tasks yielded optimal performances at the same layer, but at a higher resolution. (TIFF) [file pcbi.1012056.s006.tiff]

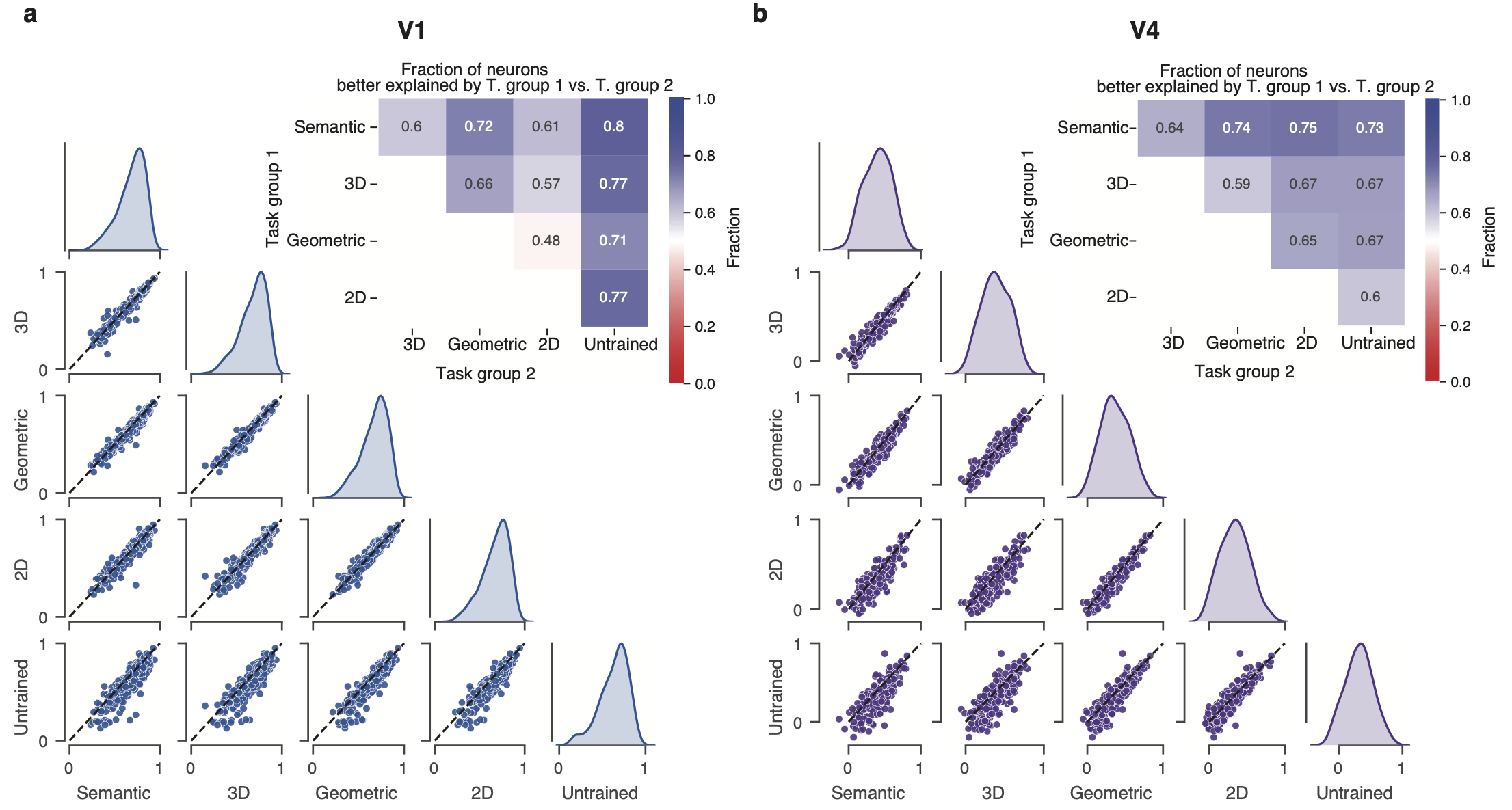

Supplement: S7 Fig — The dotted line in each pairwise comparison represents the identity and the panels in the main diagonal shows the performance distribution of each task-cluster. A pairwise Wilcoxon signed rank test reveal that the differences between task-clusters were significant (Fig 2D and 2E). Insets show the percentage of neurons better explained by one task group (row) vs another (column). (TIFF) [file pcbi.1012056.s007.tiff]
